# Content of stress granules reveals a sex difference at the early phase of cold exposure in mice

**DOI:** 10.1152/ajpendo.00317.2023

**Published:** 2023-11-22

**Authors:** Samson W. M. Cheung, Jensen H. C. Yiu, Karie T. C. Chin, Jieling Cai, Aimin Xu, Chi Ming Wong, Connie W. Woo

**Affiliations:** ^1^State Key Laboratory of Pharmaceutical Biotechnology, Li Ka Shing Faculty of Medicine, The University of Hong Kong, Hong Kong SAR, People’s Republic of China; ^2^Department of Pharmacology and Pharmacy, Li Ka Shing Faculty of Medicine, The University of Hong Kong, Hong Kong SAR, People’s Republic of China; ^3^Department of Medicine, Li Ka Shing Faculty of Medicine, The University of Hong Kong, Hong Kong SAR, People’s Republic of China; ^4^Department of Health Technology and Informatics, The Hong Kong Polytechnic University, Hong Kong, People’s Republic of China; ^5^Micon Analytics, Toronto, Ontario, Canada

**Keywords:** adaptive thermogenesis, sex dimorphism, stress granules

## Abstract

Adaptive thermogenesis is a vital physiological process for small endotherms. Female animals usually are more sensitive to cold temperature due to anatomical differences. Whether there is a sex difference at a molecular level is unclear. Stress granules (SGs) are dynamic organelles in which untranslated mRNAs reside during cellular stress. We hypothesize that the prompt response of SGs to cold stress can reveal the molecular difference between sexes. By analyzing the content in SGs of brown adipose tissue (BAT) at the early phase of cold stress for both sexes, we found more diverse mRNAs docked in the SGs in male mice and these mRNAs representing an extensive cellular reprogramming including apoptosis process and cold-induced thermogenesis. In female mice, the mRNAs in SGs dominantly were comprised of genes regulating ribonucleoprotein complex biogenesis. Conversely, the proteome in SGs was commonly characterized as structure molecules and RNA processing for both sexes. A spectrum of eukaryotic initiation factors (eIFs) was detected in the SGs of both female and male BAT, while those remained unchanged upon cold stress in male mice, various eIF3 and eIF4G isoforms were found reduced in female mice. Taken together, the unique features in SGs of male BAT reflected a prompt uncoupling protein-1 (UCP1) induction which was absent in female, and female, by contrast, were prepared for long-term transcriptional and translational adaptations.

**NEW & NOTEWORTHY** The proteome analysis reveals that stress granules are the predominant form of cytosolic messenger ribonucleoproteins of brown adipose tissue (BAT) at the early phase of cold exposure in mice for both sexes. The transcriptome of stress granules of BAT unveils a sex difference of molecular response in early phase of cold exposure in mice, and such difference prepares for a prompt response to cold stress in male mice while for long-term adaptation in female mice.

## Graphical abstract

**Figure:**
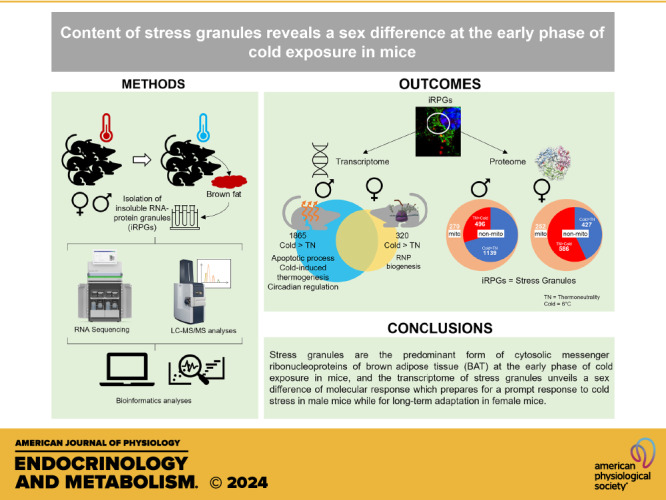

## INTRODUCTION

Thermogenetic processes including shivering and adaptive (nonshivering) thermogenesis are vital physiological processes for endotherms. Shivering thermogenesis by muscle contraction is the major heat generation among large mammals, owing to their high proportion of muscle mass (1). Small endotherms with larger surface-area-to-volume ratio, rely on adaptive thermogenesis to accommodate the greater loss of heat (2). Brown adipose tissue (BAT) is the major effector organ for adaptive thermogenesis (3) and upon cold exposure, norepinephrine secreted from sympathetic nerve acts on β-adrenergic receptors on brown adipocytes, which eventually gives rise to lipolysis and the transcription of thermogenic proteins (2). Following the activation by long-chain fatty acids, uncoupling protein-1 (UCP1) at the inner mitochondrial membrane facilitates proton transfer and dissipates the energy of substrate oxidation as heat (4). Owing to the differences in muscle and fat mass, sex presumably plays an important role in regulating different types of thermogenesis. Female animals are generally more sensitive to cold environment than males (5, 6), partially because the higher surface-area-to-volume ratio and lower muscle mass in females result in higher dissipation and lower generation of heat (7). However, besides the anatomy and physiology, the sex differences at molecular level during adaptive thermogenesis are largely unclear.

Stress granules (SGs) are cytosolic aggregates with high concentrations of RNA and protein dynamically assembled and disassembled when coping with cellular stress. They serve as a transient mRNA storage and are believed to cause translational arrest (8). An early study reported that cold temperature triggered the assembly of SGs, but it was limited to in vitro observations in which mammalian cells were cultured at 10°C (9), and it hardly resembled the physiology of endotherms. Nonetheless, cold exposure induces a robust change in transcriptome of BAT in a relatively short time (10) and it is critical to orchestrate transcription and translation to preserve energy and avoid stress-induced cellular necrosis. Here, the transcriptome and proteome of SGs in mouse BAT during early phase of cold exposure showed various molecular differences between male and female. The SGs in male BAT were enriched in genes synchronizing apoptosis and adaptive thermogenesis whereas in female BAT those were regulating ribonucleoprotein complex biogenesis. The cold-induced proteome in SGs of BAT represented structure molecules and RNA processing for both sexes. The differences of how male and female endotherms regulate resting energy metabolism via adaptive thermogenesis remind us of the importance of addressing sexual dimorphism in metabolic complications of disease conditions.

## MATERIALS AND METHODS

The detailed protocols are available in Supplemental Materials (11–15); all Supplemental material is available at https://doi.org/10.6084/m9.figshare.24556174.

## RESULTS

### Absence of Change in Thermogenic Proteins in Female BAT in the Early Phase of Cold Exposure

**Figure 1.**
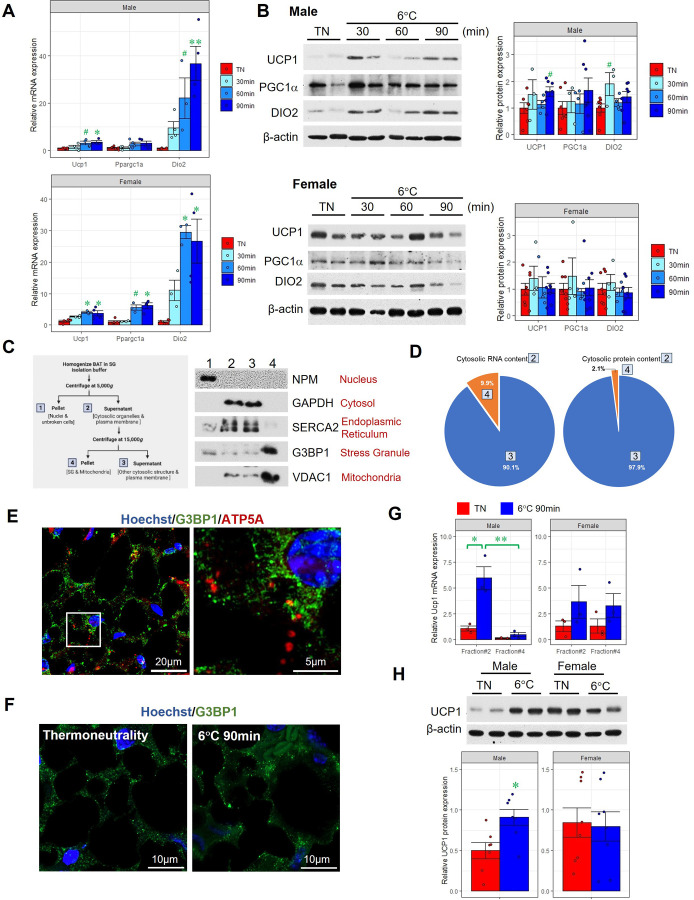
Detection of SGs marker and *Ucp1* mRNA in insoluble RNA-protein granules. Relative mRNA (*A*) and protein levels of UCP1, PGC1α, and DIO2 (*B*) in brown adipose tissue (BAT) of mice exposed to 6°C for various periods of time (*n* = 3–5 mice/time point). β-Actin was used as the loading control. *P < 0.05, **P < 0.01, #unadjusted *P* < 0.05. TN, thermoneutrality at 30°C. *C*: flowchart of isolating insoluble RNA-protein granules (iRPGs) from BAT and validation using immunoblotting of different subcellular markers. *D*: the average percentage of RNA and protein content in iRPG and non-iRPG fractions of BAT. The boxed numbers denote the fractions shown in *C*. *E*: immunofluorescence staining of stress granule marker, G3BP1, and mitochondrial marker, ATP5A, in BAT of male mice housing at 30°C using Hoechst 33342 to locate the nuclei (*n* = 3 mice for *C* to *E*). *F*: immunofluorescence staining of G3BP1 in iRPGs of male BAT (*n* = 4 mice/group). *G*: relative *Ucp1* mRNA expression in fraction no. 2 and fraction no. 4 (iRPGs) of the same prepared tissues, using TN group of female mice as basal index. *Actb* and *Fabp4* were used as housekeeping genes for whole BAT and iRPGs, respectively, based on the abundance and consistence in the fractions (*n* = 3 mice/group). *H*: immunoblotting and densitometry analysis of UCP1 protein in whole BAT (*n* = 5–8/each condition). Representative images are shown. *P < 0.05, **P < 0.01. SGs, stress granules.

Mice were housed under thermoneutral condition (30°C) since birth and exposed under 6°C for 90 min. The body temperature was dropped by 3°C–4°C in this period in both sexes (Supplemental Fig. S1*A*) but the decline in females was larger (Supplemental Fig. S1*B*). Within 90 min of cold exposure, we had not seen a robust morphological change in BAT (Supplemental Fig. S2). The mRNA of three classical thermogenesis-related genes in the BAT, *Ucp1*, peroxisome proliferator-activated receptor-γ coactivator-1α (*Ppargc1a*) and type II iodothyronine deiodinase (*Dio2*) were prominently increased starting from 60 min of cold exposure, and *Dio2* mRNA was the most robustly elevated (Fig. 1*A*). However, all these proteins were not increased within 90 min of cold exposure in female mice, and only UCP1 protein was significantly induced at 90 min exposure and a transient increase in DIO2 at 30 min exposure in male mice (Fig. 1*B*). This suggests that the sharp increase in mRNA expressions of thermogenic genes in mouse BAT upon cold exposure did not proximately reflect protein abundance, particularly for DIO2.

### Sex Difference in the Abundance of *Ucp1* mRNA Stalled in the SGs of BAT under Cold Exposure

SGs in BAT were isolated according to the previously described protocol with minor modifications (Fig. 1*C*; 11). Fractions no. 1 to no. 4 of BAT lysate were examined using various organelle markers. Fraction no. 4, which represented the heaviest material in cytosol, was enriched with Ras GTPase-activating protein-binding protein-1 (G3BP1), a marker for SGs (16), and voltage-dependent anion channel-1 (VDAC1), a marker for mitochondria (17). The RNA content in fraction no. 4 was ∼10% of that in the whole cytosol (fraction no. 2), whereas only 2% of total cytosolic proteins were found in fraction no. 4 (Fig. 1*D*). The appearance of G3BP1 as punctate in the immunofluorescent images implicated the formation of aggregates (Fig. 1*E*). Moreover, the location of G3BP1 in the cytosol was distinct from mitochondria, as indicated by the marker, ATP synthase subunit α (ATP5A; Fig. 1*E*; 18). In the following description of results, fraction no. 4 would be referred as “insoluble RNA-protein granules” (iRPGs). During the early phase of cold exposure (90 min), the abundance of G3BP1 in the iRPGs of BAT was at a similar level as those in thermoneutrality (Fig. 1*F*, Supplemental Fig. S3). When we compared *Ucp1* mRNA expression in different fractions of the same BAT samples, only a small portion was aggregated in iRPGs out of fraction no. 2 upon cold exposure in male BAT (Fig. 1*G*). The higher mRNA expression in the cytosol which favored in translation projected to the increased UCP1 protein level in male at 90 min cold exposure (Fig. 1*H*). Conversely, in female BAT, a substantial portion of *Ucp1* mRNA was detected in iRPGs (Fig. 1*G*). Although female mice had higher levels of UCP1 protein than male at thermoneutral temperature, it was not induced within 90 min of cold exposure (Fig. 1*H*). These results implicated that female mice might not need an immediate translation of UCP1 to handle the cold challenge and allowed a temporary arrest in translation by docking its transcript in iRPGs.

### Genes Regulating Apoptosis and Thermogenesis Enriched in iRPGs of Male BAT upon Cold Exposure

**Figure 2.**
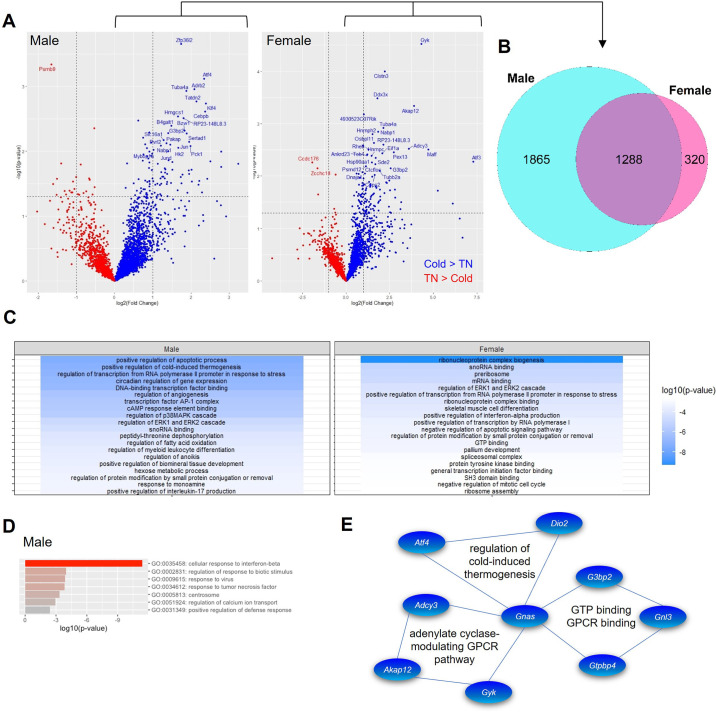
Differences in RNA content of iRPG fraction between male and female BAT. *A*: volcano plot showing fold changes in transcriptomes in iRPGs of BAT between thermoneutrality (TN) and 90-min exposure at 6°C. Genes with at least twofold changes between two conditions and an unadjusted *P* < 0.01 are annotated. *B*: Venn diagram showing number of identified genes in iRPG of BAT with abundance higher at cold exposures. Gene ontology (GO) term enrichment analysis of genes with mRNA abundance at least twofold higher in the cold exposure group with unadjusted *P* < 0.05 for male and female mice (*C*) or lower in the cold exposure for male mice (*n* = 3 mice/group; *D*). *E*: schematic depicting the interactions of genes that were commonly upregulated upon cold exposure in both sexes. BAT, brown adipose tissue; iRPG, insoluble RNA-protein granules.

Approximately 75%–90% of transcriptome in SGs is mRNA (19). We then examined the change in mRNA content in iRPGs of BAT through RNA sequencing analysis. A total of 19,823 genes were identified, and 4,945 genes were present at >10 transcripts per million (tpm) in either cold exposure or thermoneutral group in male iRPGs (Fig. 2*A*). Among these 4,945 genes, 3,153 genes were upregulated under cold exposure (Fig. 2*A*, Supplemental Table S1*A*). In contrast, in female iRPGs, 17,901 genes were identified, and 2,958 genes were present at >10 tpm in either condition, in which only 1,608 genes were upregulated under cold exposure (Fig. 2*A*, Supplemental Table S1*B*). There were 1,288 genes commonly upregulated under cold exposure for both sexes (Fig. 2*B*). The genes showing differences with unadjusted *P* < 0.05 were subjected to the functional enrichment analysis using gene ontology (GO; 13). In male mice, these cold-induced genes (119 genes) were clustered to transcription factor binding, positive regulation of apoptosis process and cold-induced thermogenesis, circadian regulation and transcription regulation from RNA polymerase II promoter in response to stress (Fig. 2*C*). The cluster of thermogenesis process included β2-adrenergic receptor (*Adrb2*), CCAAT enhancer binding protein-β (*Cebpb*), *Dio2*, G-protein stimulatory subunit α (*Gnas*), *Ppargc1a*, growth arrest and DNA damage-inducible protein-γ (*Gadd45g*), and free fatty acid receptor-4 (*Ffar4* or *Gpr120*) (Supplemental Table S2*A*). Those genes showing lower expressions under cold stress (36 genes) belonged to the clusters representing the responses to interferon-β and -γ (Fig. 2*D*, Supplemental Table S2*B*). By contrast, in female BAT, those genes in iRPGs significantly increased during cold exposure (89 genes) predominantly clustered to ribonucleoprotein complex biogenesis (Fig. 2*C*). These genes included DEAD-box helicase-3x (*Ddx3x*), *Ddx5* and *Ddx21*, eukaryotic translation initiation factor-3 subunit A (*Eif3a*), ribosomal biogenesis regulator-1 homolog (*Rrs1*), and NOP56 ribonucleoprotein (*Nop56*; Supplemental Table S2*C*). However, only three genes in iRPGs of female BAT were significantly higher in thermoneutrality than cold exposure (Supplemental Table S2*D*). On the other hand, among those genes showing differences with unadjusted *P* < 0.05, 27 cold-induced genes in the iRPGs were commonly present in BAT of both sexes, and they were clustered to G-protein-coupled receptor binding and cold-induced thermogenesis regulation (Fig. 2*E*).

### No Distinctive Change in iRPG Proteomes of BAT for Both Sexes upon Cold Exposure

**Figure 3.**
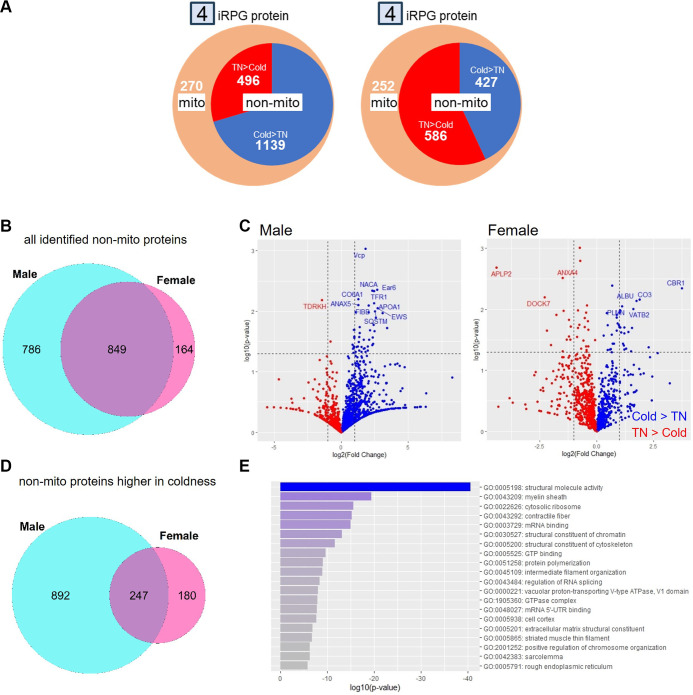
Common proteome in iRPGs of BAT from both sexes. *A*: Pie charts showing the numbers of identified proteins of BAT between thermoneutrality (TN) and 90-min exposure at 6°C. Mito, mitochondrial proteins. Red indicating the portion of proteins showing higher abundance under TN and blue indicating those higher under cold exposure. *B*: Venn diagram showing number of identified nonmitochondrial protein iRPG of BAT. *C*: volcano plot showing fold changes in proteomes in iRPGs of BAT between TN and 90-min cold exposure. Proteins with at least twofold changes between two conditions and an unadjusted *P* < 0.01 are annotated. *D*: Venn diagram showing number of identified nonmitochondrial protein iRPG of BAT that were higher in cold condition. *E*: gene ontology (GO) term enrichment analysis of nonmitochondrial proteins commonly increased by cold exposure for both sexes (*n* = 3 mice/group). BAT, brown adipose tissue; iRPG, insoluble RNA-protein granules.

The protein content of iRPGs in the BAT was examined using LC-MS/MS-based proteomics analysis. In male mice, 1,905 proteins were identified in the iRPGs, among which 270 were mitochondrial proteins, given the fact that the method of iRPG isolation could not exclude mitochondria (Fig. 3*A* and Fig. 2*A*). There were 1,635 nonmitochondrial proteins in the iRPGs, in which 1,139 proteins were higher and 496 proteins were lower in cold exposure than in the thermoneutral group (Fig. 3*A*). In contrast, in female mice, only 1,265 proteins were identified in the iRPGs, among which 252 were mitochondrial proteins, the proportion similar to that in male mice (Fig. 3*A*). These mitochondrial proteins shared substantial similarity between sexes (Supplemental Fig. S4 and Supplemental Table S3). In the 1,013 nonmitochondrial proteins in the iRPGs of female mice, 427 proteins were higher, and 586 proteins were lower in cold exposure than in the thermoneutral group (Fig. 3*A*). Many of the identified nonmitochondrial proteins in the iRPGs of female BAT were also found in male BAT (Fig. 3*B*). Among the nonmitochondrial proteins showing difference between thermoneutral and cold conditions with unadjusted *P* < 0.05, 48 out of 52 proteins were higher under cold condition in male whereas only 35 out of 68 proteins in female (Fig. 3*C*, Supplemental Table S4). Furthermore, there were 247 proteins commonly showing a trend of increase upon cold exposure between male and female BAT (Fig. 3*D*). These were mainly clustered into pathways representing structure molecule activity and various RNA processing in the enrichment analysis (Fig. 3*E*, Supplemental Table S5).

### SGs as Dominant Messenger Ribonucleoprotein Aggregates in the iRPGs of BAT

**Figure 4.**
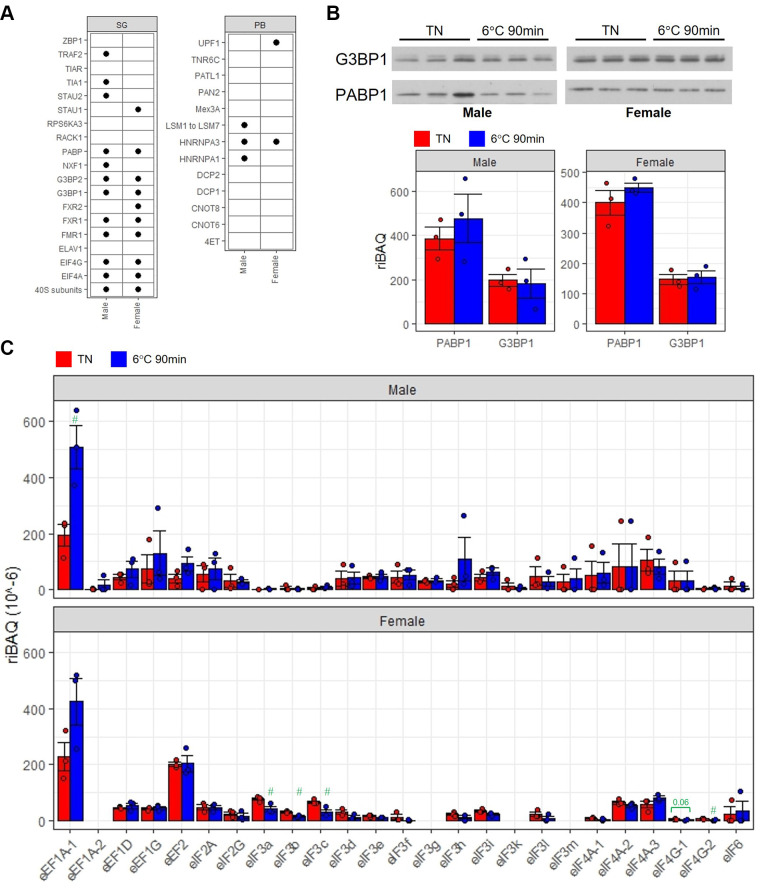
The presence of SG markers in iRPG of BAT in both sexes. *A*: the presence (•) of SG or P-bodies (PB) markers in iRPG fraction of BAT. *B*: immunoblotting for G3BP1 and PABP1 (*top*) and their readout of proteomics analysis (*bottom*) in the iRPG of BAT under thermoneutrality (TN) and 90-min exposure at 6°C (*n* = 3 mice/group). *C*: protein abundance of eukaryotic elongation factor (eEF) and initiation factor (eIF) isoforms detected in iRPGs of BAT in the unit of relative intensity-based absolute quantification (riBAQ) by proteomics analysis. #unadjusted *P* < 0.05 (*n* = 3 mice/group). BAT, brown adipose tissue; iRPG, insoluble RNA-protein granules; SG, stress granule.

Cytosolic messenger ribonucleoproteins (mRNP) can be in the form of SGs and P-bodies, and unlike SGs where translation initiation components dock, P-bodies contain mRNA decay machinery (20). SGs and P-bodies are distinctive structures but can interact to exchange material to regulate mRNA metabolism (20). The iRPGs of BAT contained many of the SGs markers but only a few of P-bodies markers for both sexes (20, 21; Fig. 4*A*). It is possible that the docked mRNA has yet directed to the decay pathway at the early stage of cold exposure, which allows the timely response in translation when coldness persists. We also examined two classical SG markers, G3BP1 and PABP1 in the iRPGs of BAT using Western immunoblotting (Fig. 4*B*). No difference in the abundance of G3BP1 and PABP1 between thermoneutral and cold conditions and between male and female BAT was observed, which were similar to the data of proteomics analysis (Fig. 4*B*). Unlike the transcriptome, the common proteome of iRPGs in BAT of both sexes suggests that the assembly and stabilization of SGs rely on certain core proteins. In eukaryotic cells, a spectrum of translation complex proteins often docks along with the mRNA in SGs so that they can be readily translated (20, 21). We examined whether there were changes in the translation complex proteins in iRPG upon cold challenge. Various types of translation complex proteins including eukaryotic initiation factors (eIFs), eIF2, 3, 4, and 6 and elongation factors (eEFs), eEF1 and 2 were detected in the iRPGs of BAT for both sexes from the proteomics analysis (Fig. 4*C*). In male, there was no difference in the detected eIFs and eEFs in iRPGs of BAT, except eEF1A1, between cold and thermoneutral conditions (Fig. 4*C*). By contrast, in females, four of the eIFs, including eIF3a, eIF3b, eIF3b, and eIF4G2, were significantly lower in iRPGs under cold exposure than in thermoneutrality (Fig. 4*C*). Only the antibodies for eIF4G were sensitive enough to detect the expressions in iRPGs and the Western immunoblots showed lower expressions for both eIF4G1 and eIF4G2 in cold than thermoneutral conditions in the iRPGs of female BAT (Supplemental Fig. S5). Take together, the lower diversity of proteome (Fig. 3*A*) and eIF3s and eIF4Gs expressions (Fig. 4*C*) suggested a lower amount of SGs in BAT of female mice upon cold challenge when compared with males. Data supplements can be accessed here: https://doi.org/10.6084/m9.figshare.24556174.

## DISCUSSION

Adaptive thermogenesis is a crucial physiological process for small animals. After *Ucp1* mRNA being transcribed in the nucleus and translated in the cytosol, the de novo synthesized UCP1 protein is translocated into the inner mitochondrial membrane where it facilitates proton leakage across the membrane and subsequently heat generation (4). When Ucp1 mRNA is sequestered in SGs in the cytosol, such aggregation becomes a handy hub and regulates its translation during cold stress (Fig. 1*G*). In fact, a higher basal UCP1 expression in BAT was observed in female mice compared with male (Supplemental Fig. S4), and such higher protein level forestalled an immediate translation of *Ucp1* mRNA in female mice upon cold challenge, allowing its docking in SGs. Although the precise localization of SGs is context-dependent, it is conceivable that certain mRNAs involved in thermogenesis can be present in SGs in the cytosol. The subcellular localization of proteins and mRNAs involved in thermogenesis appears to be relevant to their functions and physiological expressions.

Male BAT in general shows a wider variety of transcriptome and proteome in SGs compared with female BAT upon cold exposure, suggesting more extensive reprogramming of BAT in male mice. Indeed, the enrichment analysis revealed not only genes responsible for cold-induced thermogenesis were upregulated but also those for apoptosis and circadian regulations (Fig. 2*C*). The enrichment of ribonucleoprotein complex biogenesis in female mice is likely for handling subsequent increase in general transcription and translation. Although in human, there is no difference in resting energy expenditure between sexes upon short-term cold exposure, females show a more prominent drop in plasma glucose and leptin but a rise in adiponectin, suggesting that endocrinological responses upon coldness are crucial in females (22). The molecular response to cold exposure may designate for comprehensive physiological adaptations instead of immediate thermogenesis in female. On the other hand, those genes that become less in the iRPGs of male BAT upon cold exposure were clustered in regulation of viral response and interferon pathways (Fig. 2*C*). This observation aligns with a previous report that PRDM16, a transcription factor activating BAT-specific genes, represses type I interferon response (23), and interferon regulatory factor-3 (IRF3) is a strong repressor for thermogenic genes (24). The reason why these immunological genes are transcribed in BAT at basal condition requires further investigation.

The mixture of mitochondrial proteins in the iRPGs fraction is one of the limitations of our study. The separation of iRPGs from cytosol relies on the weight of such protein aggregates, but the similar density of mitochondria prevents further separation. An alternative method such as immunoprecipitation has been used to separate mitochondria from the iRPGs (25). However, the additional protein interactions may trigger a partial disassembly of aggregates, and the purified content is limited to those bound to the chosen antigens. Bioinformatics approach to exclude mitochondrial proteins allowed us to analyze the transcriptome and proteome in SGs of BAT comprehensively. Another limitation was the lack of precise quantification of SGs. Because SGs are dynamic structures, the detection and quantification of organelle markers such as G3BP1 does not absolutely reflect the abundance of total SGs. However, the higher diversity of mRNA in iRPG of male BAT upon cold exposure indirectly suggests a higher amount of SGs because mRNA is the driving force of SG assembly (26). The lower abundance of several eIF isoforms, which are the key components of SGs, and diversity of proteome in iRPGs of female BAT likely implicates a lower amount of SGs (20).

In conclusion, the content of SGs in BAT reveals that males and females respond differently to the early phase of cold exposure at molecular level in mice. It is noteworthy to investigate whether such sex difference is also present in the beige adipocytes of larger endotherms such as human, which may suggest molecular differences at basal metabolism.

## DATA AVAILABILITY

RNA-Seq data are available in GenBank under accession number PRJNA991637. Proteomics data are available at PRIDE under accession number PXD044433. Other data supporting the findings of this study are available from the corresponding author upon reasonable request.

## SUPPLEMENTAL DATA

10.6084/m9.figshare.24556174 Supplemental Material, Supplemental Figs. S1–S5, and Supplemental Tables S1–S6: https://doi.org/10.6084/m9.figshare.24556174.

## GRANTS

University Grants Committee (UGC), Research Grant Council (RGC) Hong Kong, Grant Nos.: GRF17101118 and GRF17102920 (to C.W.W.), AoE/M-707/18 (to A.X. and C.W.W.). University Grants Committee (UGC), Research Grant Council (RGC) Hong Kong, Hong Kong RGC Postdoctoral Fellowship Scheme, Award Nos.: PDFS 2021-7S06 (to J.H.C.Y.).

## DISCLOSURES

No conflicts of interest, financial or otherwise, are declared by the authors.

## AUTHOR CONTRIBUTIONS

C.W.W. conceived and designed research; S.W.M.C., J.H.C.Y., K.T.C.C., and J.C. performed experiments; S.W.M.C., J.H.C.Y., and C.W.W. analyzed data; S.W.M.C., J.H.C.Y., and C.W.W. interpreted results of experiments; S.W.M.C., J.H.C.Y., and C.W.W. prepared figures; S.W.M.C. and C.W.W. drafted manuscript; S.W.M.C., J.H.C.Y., A.X., C.M.W., and C.W.W. edited and revised manuscript; J.H.C.Y., K.T.C.C., C.M.W., and C.W.W. approved final version of manuscript.
